# Caveolae compartmentalise β2-adrenoceptor signals by curtailing cAMP production and maintaining phosphatase activity in the sarcoplasmic reticulum of the adult ventricular myocyte

**DOI:** 10.1016/j.yjmcc.2011.06.014

**Published:** 2012-02

**Authors:** David A. MacDougall, Shailesh R. Agarwal, Elizabeth A. Stopford, Hongjin Chu, Jennifer A. Collins, Anna L. Longster, John Colyer, Robert D. Harvey, Sarah Calaghan

**Affiliations:** a Institute of Membrane and Systems Biology, University of Leeds, Leeds, LS2 9JT, UK; b Department of Pharmacology, University of Nevada School of Medicine, Reno, NV 89557, USA

**Keywords:** AKAP, A kinase anchoring protein, ARVM, adult rat ventricular myocyte, β-AR, beta adrenoceptor, C3SD, caveolin-3 scaffolding domain, cAMP, cyclic adenosine 3′-5′-monophosphate, Cav-3, caveolin-3, CGP, CGP20712A, EHNA, *erythro*-9-(2-hydroxy-3-nonyl)adenine, Epac, exchange protein activated by cAMP, FRET, fluorescence resonance energy transfer, FSK, forskolin, HIV, human immunodeficiency virus, IBMX, 3-isobutyl-1-methylxanthine, *I*_Ca,L_, L-type Ca^2+^ current, Iso, isoproterenol bitartrate, MBCD, methyl-β-cyclodextrin, PDE, phosphodiesterase, PKA, protein kinase A, PLB, phospholamban, pPLB, Ser^16^ phosphorylated PLB, PP, protein phosphatases, RyR, ryanodine receptor, TAT, trans-activating transcriptional activator, TnI, troponin I, ZNT, zinterol hydrochloride, Caveolae, Cyclic AMP, Compartmentation, β2-adrenoceptor, Phosphatase

## Abstract

Inotropy and lusitropy in the ventricular myocyte can be efficiently induced by activation of β1-, but not β2-, adrenoceptors (ARs). Compartmentation of β2-AR-derived cAMP-dependent signalling underlies this functional discrepancy. Here we investigate the mechanism by which caveolae (specialised sarcolemmal invaginations rich in cholesterol and caveolin-3) contribute to compartmentation in the adult rat ventricular myocyte. Selective activation of β2-ARs (with zinterol/CGP20712A) produced little contractile response in control cells but pronounced inotropic and lusitropic responses in cells treated with the cholesterol-depleting agent methyl-β-cyclodextrin (MBCD). This was not linked to modulation of L-type Ca^2+^ current, but instead to a discrete PKA-mediated phosphorylation of phospholamban at Ser^16^. Application of a cell-permeable inhibitor of caveolin-3 scaffolding interactions mimicked the effect of MBCD on phosphorylated phospholamban (pPLB) during β2-AR stimulation, consistent with MBCD acting via caveolae. Biosensor experiments revealed β2-AR mobilisation of cAMP in PKA II signalling domains of intact cells only after MBCD treatment, providing a real-time demonstration of cAMP freed from caveolar constraint. Other proteins have roles in compartmentation, so the effects of phosphodiesterase (PDE), protein phosphatase (PP) and phosphoinositide-3-kinase (PI3K) inhibitors on pPLB and contraction were compared in control and MBCD treated cells. PP inhibition alone was conspicuous in showing robust de-compartmentation of β2-AR-derived signalling in control cells *and* a comparatively diminutive effect after cholesterol depletion. Collating all evidence, we promote the novel concept that caveolae limit β2-AR-cAMP signalling by providing a platform that not only attenuates production of cAMP but also prevents inhibitory modulation of PPs at the sarcoplasmic reticulum. This article is part of a Special Issue entitled “Local Signaling in Myocytes”.

## Highlights

► We investigate the mechanism(s) by which caveolae compartmentalise β2-AR signalling. ► Disrupting caveolae reveals β2-AR cAMP signals in the PKA-RII compartment. ► Disrupting caveolae promotes a selective PKA-mediated phosphorylation of PLB. ► Phosphatases (PP) 1/2a (but not PDE 2/3/4) contribute to caveolar β2-AR compartmentation. ► We propose that intact caveolae attenuate cAMP production and limit inhibitory modulation of PP at the SR.

## 1 Introduction

In the cardiac myocyte, many receptors signal via the second messenger cyclic AMP, but produce diverse changes in electrical, mechanical, metabolic and transcriptional activities. This occurs because different receptors produce changes in cAMP in different compartments of the cell. The idea of cAMP compartmentation is well accepted but the mechanisms responsible are not fully understood.

β1- and β2-adrenoceptors (AR), the predominant β-ARs in the heart, provide an excellent illustration of compartmentalised cAMP signalling in the adult ventricular myocyte. β1-AR stimulation promotes positive inotropic and lusitropic responses, whereas β2-AR stimulation has minimal functional effects [1,2]. These distinct β-AR functional responses can be correlated with temporal and spatial properties of cAMP generation [3], protein kinase A (PKA) activation [4] and target protein phosphorylation [5]. In essence, β1-AR signals are global, whereas β2-AR signals are localised to their site of production. For example, β1-AR stimulation promotes PKA-dependent phosphorylation of multiple targets throughout the cell — the L type Ca^2+^ channel [6], phospholamban (PLB) [7], RyR [8] and troponin I (TnI) [7]. By contrast, β2-AR signals do not access proteins of the sarcoplasmic reticulum (SR) or myofilaments [9,10].

We are interested in the mechanisms that restrict cAMP dependent signalling from the β2-AR.

Whilst the β1-AR couples to the stimulatory Gs protein, β2-ARs couple sequentially to Gs and the inhibitory Gi protein. The Gi pathway has been proposed as a key factor responsible for the spatial restriction of β2-AR-cAMP signals to the sarcolemmal compartment, perhaps through stimulation of protein phosphatase activity [11–13]. Another focus of interest is phosphodiesterase (PDE). Several studies have suggested that PDE 3 and 4 contribute to the creation of the distinct β2 AR cAMP signatures in the adult ventricular myocyte, because PDE3/4 inhibition increases the size and duration of the cAMP and activated PKA signals (e.g. [4,14]).

However, an additional, complementary, explanation for the diverse effects of β1- and β2-AR stimulation on cAMP is that the differential membrane distribution of signalling elements, including receptors, produces cAMP signals in different cellular compartments. The lipid bilayer is not a homogenous structure with randomly distributed proteins, but contains liquid-ordered phases enriched in cholesterol and sphingolipids known as lipid rafts. Caveolae are specialised forms of invaginated rafts characterised by the presence of caveolin and cavin proteins (see Refs. [15,16] for reviews). Caveolae have been shown to be important structures for organising G-protein coupled receptors and their downstream signalling components [17]. Within caveolae, oligomeric caveolin can assemble, and regulate, macromolecular signalling complexes [18] through its ability to bind many different proteins via its scaffolding domain [19].

In support of a role for caveolae in creating distinct β1- and β2-AR cAMP signals, differential membrane distribution of receptors has been reported in the ventricular myocyte. Whilst β1-ARs are found in caveolar and non-caveolar domains, the majority of β2-ARs are present exclusively in caveolae (in the absence of β AR stimulation) [20–23]. Other elements of the β AR signal cascade (Gαs, Gαi, adenylyl cyclase 5/6, PKA) have been shown to be present in caveolar membrane fractions [20–23], to co-immunprecipitate with caveolin 3 [22] and to be inhibited by interaction with the caveolin scaffolding domain [24–26]. The exclusive caveolar location of the β2-AR suggests a role for caveolae in its downstream signalling. Indeed we have recently shown functional evidence that caveolae compartmentalise the β2 AR response in the adult ventricular myocyte; disruption of caveolae reveals marked inotropic and lusitropic responses to β2-AR stimulation [27], associated with a globalisation of cAMP-dependent signalling indexed by phosphorylation of phospholamban [9].

The aim of the present study was to reconcile, for the first time, work showing the roles of phosphatases and phosphodiesterases in spatial control of β2-AR signalling with mechanism(s) by which caveolae compartmentalise cAMP in the adult ventricular cell. We report how disruption of caveolae impacts on β2-AR signalling at the level of cAMP, protein phosphorylation, *I*_Ca,L_, [Ca^2+^]_i_ and contraction, and how this is affected by inhibition of key components linked with β2-AR compartmentation. Measuring these different indices gives insight into the mechanism of caveolar control (e.g. cAMP production/cAMP degradation/phosphatase activity) and the region of the cell (e.g. sarcolemma/SR/myofilaments) in which this control operates. We show that spatial restriction of the β2-AR cAMP signal by caveolae occurs at the level of cAMP production *and* phosphatase activity in the SR. This can be explained by the formation of specific signalling complexes in the caveolar microdomain.

## 2 Materials and methods

### 2.1 Cell isolation

Adult rat ventricular myocytes (ARVM) were enzymatically isolated from the hearts of male Wistar rats using a standard procedure outlined elsewhere [28]. Care was taken to follow the *Animals (Scientific Procedures) Act 1986*, the *Recommendation from the Declaration of Helsinki* and the *Guiding Principles in the Care and Use of Animals*. All experiments were carried out at room temperature unless otherwise stated and, except for those involving fluorescence resonance energy transfer (FRET), used ARVM on the day of isolation in an extracellular buffer containing (mM): NaCl 137, KCl 5.4, MgCl_2_ 0.5, CaCl_2_ 1.0, NaH_2_PO_4_ 0.33, glucose 5.5, HEPES 5 (pH 7.4).

### 2.2 Cell culture

For FRET imaging experiments only, ARVM were plated in minimum essential medium (MEM) containing insulin–transferrin–selenium complex (Invitrogen, CA, USA), bovine serum albumin (1 mg/ml), 2,3-butanedione monoxime (10 mM) and penicillin–streptomycin. After incubation for 2 h, the cells were transduced with adenoviral constructs containing protein kinase A (PKA)- or exchange protein activated by cAMP (Epac)-based cAMP biosensors, as described previously [23,29,30]. Imaging experiments were carried out 48–72 h after transduction.

### 2.3 β2-AR stimulation

Selective β2-AR stimulation was achieved with the β2 agonist zinterol (ZNT, 10 μM) in combination with CGP20712A (CGP, 300 nM), a specific β1-AR antagonist. For all protocols, cells were exposed to CGP for at least 5 min prior to addition of solution containing ZNT and CGP. Baseline recordings were made in the presence of CGP.

### 2.4 Methyl-β-cyclodextrin treatment

Freshly isolated ARVM were incubated for 1 h at 37 °C in Ca^2+^-containing isolation solution (Ca-IS, [in mM]: NaCl 130, KCl 5.4, MgCl_2_ 1.4, NaH_2_PO_4_ 0.4, creatine 10, taurine 20, glucose 10, HEPES 5, (pH 7.4)) supplemented with 1–2 mM methyl-β-cyclodextrin (MBCD); cultured cells were incubated under the same conditions with MBCD dissolved in MEM-based culture medium. MBCD solution was replaced with fresh solution and treated cells used within 3 h. We have previously shown that incubation of myocytes with 1–2 mM MBCD at 37 °C for 1 h removes cholesterol and Cav-3 from buoyant caveolar membrane fractions [9] and markedly reduces caveolae number [31].

### 2.5 TAT-tagged peptide treatment

Peptides corresponding to the 11-residue trans-activating transcriptional activator sequence (TAT) from HIV-1, N-terminally linked via 4 glycine residues to the 20-residue caveolin scaffolding domain of Cav-3 (C3SD) or a scrambled version of this (Scram) were synthesised (Peptide 2.0, VA, USA). Complete peptide sequences were: TAT-C3SD, YGRKKRRQRRRGGGGDGVWRVSYTTFTVSKYWCYR; TAT-Scram, YGRKKRRQRRRGGGGYWTVYTKVDFCGSRYVRTSW. Lyophilised stock aliquots (0.5 mg) were reconstituted in water and diluted in Ca-IS. ARVM were incubated in 0.5 or 1 μM peptide-Ca-IS solution at 37 °C for 30 min. Peptide solutions ≥ 2 μM caused a marked decrease in the viability of myocyte preparations.

### 2.6 Cell shortening and [Ca^2+^]_i_ transient measurements

Unloaded shortening and [Ca^2+^]_i_ transients were recorded simultaneously using digital edge-detection software (IonOptix, MA, USA) and an OptoScan monochromator (Cairn Research, UK) in field-stimulated ARVM (0.5 Hz) loaded with 1.5 μM fura-2, following previously described protocols (Calaghan et al., 1998). An average of 10–12 traces under basal conditions, and at steady-state following drug application, were analysed using IonWizard 6.0 software (IonOptix).

### 2.7 *I*_Ca,L_ measurement

*I*_Ca,L_ was recorded as described in Agarwal et al. [23]. In brief, experiments were performed using Cs^+^-based extracellular and intracellular solutions in the whole-cell configuration. Membrane potential was stepped from − 80 mV to − 40 mV (50 ms) to inactivate *I*_Na_, followed by a 100 ms step to 0 mV to activate *I*_Ca,L_. This protocol was repeated at 5 s intervals.

### 2.8 FRET imaging

Experiments were performed using myocytes expressing PKA- or Epac2-based sensors as described previously in detail [23,29,30]. Changes in cAMP levels were defined as relative changes in the ratio of the background and bleed-through corrected CFP and YFP (FRET) fluorescence intensity measured over the area of the entire cell.

### 2.9 Total and phospho-protein detection

Myocyte populations used to measure phospho-protein responses to β2-AR stimulation were field-stimulated (0.5 Hz) during drug treatment, then immediately homogenised in immunoblot sample buffer (ISB: 62.5 mM Tris [pH 6.8], 10% [^v^/_v_] glycerol, 2% [^w^/_v_] SDS, 1x protease (Roche, Basel, Switzerland) and phosphatase (Thermo Fisher Scientific, Northumberland, UK) inhibitor cocktails). Samples were immediately frozen and stored at − 20 °C until required; 5% (^v^/_v_) β-mercaptoethanol was added and samples heated to 95 °C for 5 min before electrophoresis. Proteins were separated by SDS-PAGE, transferred to PVDF membranes and antibody steps were performed as described in Calaghan et al. [28]. Loading volumes were standardised to rod-shaped cell number, depending on the target (5000 cells/well for total and phosphorylated PLB; 30 000 cells/well for total and phosphorylated TnI and RyR2). Following enhanced chemiluminescence, only non-saturated signals were quantified (Aida Image Analyzer).

### 2.10 Membrane fractionation

ARVM membrane populations were fractionated in a detergent-free system using a discontinuous sucrose density gradient as recently described [9]. Caveolae-containing fractions (5 and 6) were identified on the basis of high buoyant density, enrichment in Cav-3 and exclusion of β-adaptin.

### 2.11 Materials

All materials were purchased from Sigma-Aldrich (Dorset, UK) unless otherwise stated. Zinterol hydrochloride was from Tocris Bioscience (MO, USA) and EHNA (*erythro*-9-(2-hydroxy-3-nonyl)adenine) hydrochloride was from Calbiochem (NJ, USA). Fura-2AM was from Molecular Probes. β2-AR, Gαi3, AC5/6, and GRK2 antibodies were obtained from Santa Cruz Biotechnology (CA, USA). PLB, PLB phospho-Ser^16^, RyR phospho-Ser^2809^ and RyR phospho-Ser^2030^ were from Badrilla (West Yorkshire, UK) [32]. β-adaptin and monoclonal Cav-3 antibodies were all from BD Transduction Laboratories (NJ, USA). PP2A, TnI, and TnI phospho-Ser^23/24^ antisera, calyculin-A, milrinone and rolipram were purchased from Cell Signaling Technology (MA, USA). The PKA-RII antibody was from Millipore (MA, USA). The PDE3A antibody was purchased from FabGennix (TX, USA). The PDE4D antibody was a kind gift from Prof. Miles Houslay (University of Glasgow, UK) [33,34].

### 2.12 Statistical analysis

All data are expressed as mean ± S.E.M of n preparations. Statistical significance (P < 0.05) was determined by the Student's t-test.

## 3 Results

In the last decade, studies of the role of caveolae in cAMP signalling in the ventricular myocyte have been conducted primarily in neonatal cells [22,35–37]. However, this is not the perfect model of its adult counterpart as developmental changes in cAMP compartmentation occur in these cells [20]. Therefore from the outset our aim was to conduct all our experiments in a single physiologically relevant system, the adult ventricular myocyte.

**Fig. 1:**
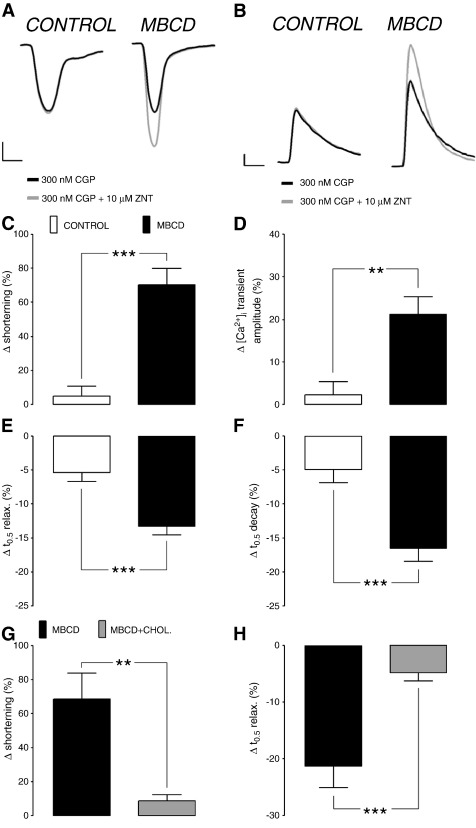
Cholesterol depletion uncovers marked β2-AR-induced inotropy and lusitropy in adult rat ventricular myocytes (ARVM). Representative myocyte shortening (A) and [Ca^2+^]_i_ transient (B) traces from individual control and MBCD-treated cells under basal conditions (300 nM CGP) and after β2-AR activation with 10 μM ZNT + 300 nM CGP. Scale bars represent 2% and 5% change from baseline (y-axes in A and B, respectively) and 200 ms (x-axes). Mean data show ZNT-mediated change (Δ) in shortening (C), [Ca^2+^]_i_ transient amplitude (D), time to half (t_0.5_) relaxation (E), time to half (t_0.5_) decay of [Ca^2+^]_i_ transient (F) in control and MBCD-treated ARVM (n = 12–20 cells from > 3 animals; **P < 0.01, ***P < 0.001). ZNT-mediated change in shortening (G) and t_0.5_ relaxation (H) in MBCD- and MBCD/cholesterol complex-treated ARVM (n = 11–13 cells from > 3 animals; **P < 0.01, ***P < 0.001). All comparisons with Student's t-test.

We began by looking at the effect of disrupting caveolae with MBCD on the functional response to β2-AR stimulation. We have previously used a combination of the β2-AR agonist salbutamol and the β1-AR antagonist atenolol to activate the β2-AR pathway in the ARVM. For the present study, we chose the more selective β2-AR agonist zinterol [38], in combination with CGP20712A, a specific β1-AR antagonist. As shown in Figs. 1 (A–D), we saw no significant change in shortening (4.9 ± 5.8%) or [Ca^2+^]_i_ transient amplitude (2.2 ± 3.1%) upon application of ZNT in the presence of CGP in control myocytes. By contrast, MBCD-treated ARVM showed a robust inotropic response to ZNT application (70.2 ± 9.7%) associated with a corresponding increase in the amplitude of the [Ca^2+^]_i_ transient (21.1 ± 4.1%). A small positive lusitropic response (5.4 ± 1.3% decrease in time to half relaxation, *t*_0.5_ relax) and increase in rate of [Ca^2+^]_i_ transient decay (5.0 ± 1.9% decrease in *t*_0.5_ decay) were seen with β2-AR stimulation in control cells; this was markedly enhanced (P < 0.001) in MBCD-treated ARVMs (to 13.3 ± 1.3% decrease in *t*_0.5_ relax and 16.5 ± 1.9% decrease in *t*_0.5_ decay)(Figs. 1E, F). Thus, β2-AR stimulation does not manifest measurable inotropic responses in control ARVM but elicits robust inotropic and lusitropic responses in ARVM depleted of cholesterol, which are associated with corresponding changes in [Ca^2+^]_i_.

The effects of MBCD can be ascribed solely to its ability to scavenge cholesterol (rather than any non-specific actions of such a large oligosaccharide), as the inotropic and lusitropic responses to β2-AR stimulation were attenuated (P < 0.001 and P < 0.01, respectively) by conjugating 1 mM MBCD with cholesterol in a molar ratio of 1:8 (Figs. 1G, H).

**Fig. 2:**
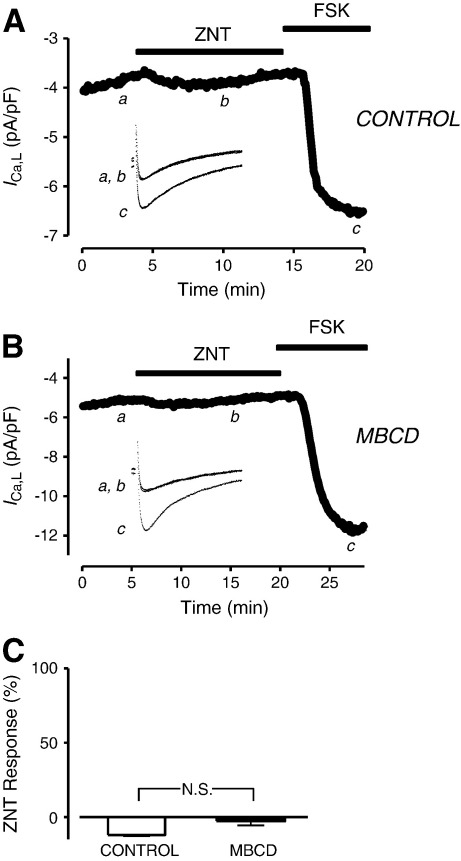
β2-AR activation does not modulate the L-type Ca^2+^ current (*I*_Ca,L_) in either control or cholesterol-depleted ARVM. Time course of changes in *I*_Ca,L_ amplitude and corresponding sample current traces (inset) under control conditions (300 nM CGP, a), following exposure to 10 μM ZNT + 300 nM CGP (b), and after exposure to 1 μM forskolin (FSK, c) in a control cell (A) and MBCD-treated cell (B). C, average ZNT-mediated increase in *I*_Ca,L_ amplitude (n = 3–4; N.S. = not significant; Student's t-test). MBCD-treatment had no effect on basal *I*_Ca,L_ density (− 5.1 ± 0.26 vs *−* 5.7 ± 0.31 pA/pF in control and MBCD-treated ARVM respectively) as reported previously [27] (n = 17; P > 0.05; Student's t-test).

Recent studies have suggested that the cAMP signals generated by β2-AR activation are small in magnitude and transient in nature [3,4]. It follows that only very proximal targets might be susceptible to β2-AR derived PKA phosphorylation. The β2-AR and the L-type Ca^2+^ channel have been shown to be closely associated in caveolar microdomains in the ARVM [22,39]. We therefore reasoned that *I*_Ca,L_ was a likely candidate for the observed functional consequences of MBCD treatment. However, as Fig. 2 shows, ZNT had no significant effect on *I*_Ca,L_ in either control or MBCD-treated ARVMs, although both groups showed robust increases in current after application of the adenylyl cyclase (AC) activator forskolin (FSK). These data suggest that the β2-AR contractile effects emerging after cholesterol depletion are not due to modulation of *I*_Ca,L_.

**Fig. 3:**
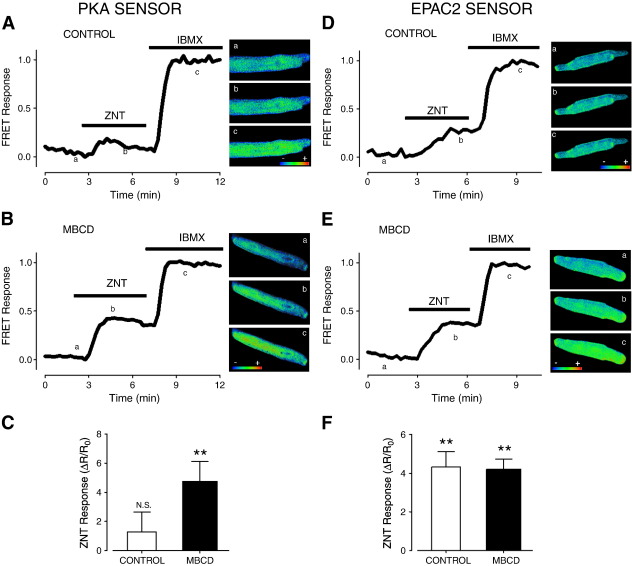
β2-AR stimulation elicits a cAMP response detected by the type II PKA-based biosensor in MBCD-treated, but not control, ARVM. Time course of changes in FRET response (ΔR/R_0_) and corresponding pseudocolor images recorded using the PKA sensor under control conditions (i.e. 300 nM CGP, a), and following exposure to 10 μM ZNT + 300 nM CGP (b) and 1 μM Iso + 100 μM IBMX (c) in a control (A) and MBCD-treated (B) cell. C, average ZNT-mediated change in FRET responses in untreated and MBCD-treated cells. Time course of changes in FRET response (ΔR/R_0_) and corresponding pseudocolor images recorded using the cytosolic Epac2-based biosensor under control conditions (i.e. 300 nM CGP, a), and following exposure to 10 μM ZNT + 300 nM CGP (b) and 1 μM Iso + 100 μM IBMX (c) in a control (D) and MBCD-treated (E) cell. F, average ZNT-mediated change in FRET responses in control and MBCD-treated cells (n = 6−13 cells; **P < 0.05, N.S. = not significant; 1-sample t-test).

Next we sought to describe the temporal and spatial characteristics of the cAMP signal underlying the robust inotropic and lusitropic response to β_2_-AR activation in cholesterol-depleted ARVM using different FRET-based cAMP biosensors. Experiments using biosensors necessitated the maintenance of cells in culture for 48–72 h. Although we have previously reported a reduction in total cellular cholesterol in ARVM cultured for 72 h, MBCD is still effective in reducing cellular cholesterol in these cells [23]. Furthermore, we are confident that culture does not result in a marked loss of caveolae at the cell surface (suggesting that cholesterol may not be depleted specifically from caveolar domains) (see Supplementary Fig. 1). We began by using an engineered cAMP sensor based on an intact PKA holo-enzyme containing only type II regulatory (RII) subunits. Through its RII-based design, this sensor is localised to specific subcellular locations via interactions with A-kinase anchoring proteins (AKAPs). These locations include caveolar and non-caveolar sarcolemma and SR membranes [35,40]. In control ARVM expressing the PKA-based biosensor, exposure to 10 μM ZNT produced no significant change (P > 0.05) in the FRET response from baseline (1.3 ± 1.4%) (Fig. 3C), although in 3/6 cells we saw a small transient response (as illustrated in Fig. 3A). All cells showed a robust PKA-probe response to 1 μM isoproterenol with 100 μM isobutyl-1-methylxanthine (IBMX). When the same experiments were performed with MBCD-treated ARVM, we saw a significant increase in the FRET response to ZNT (4.7 ± 1.4%) (Figs. 3B, C). In 7/13 cells this response was sustained during the 5 min ZNT exposure (as illustrated in Fig. 3B). We have therefore detected, in real-time, a β2-AR mobilisation of cAMP in PKA-II containing regions of the MBCD-treated ARVM that is not evident in control cells. These data mirror the effects of MBCD on shortening and [Ca^2+^]_i_ transient amplitude responses to ZNT.

In subsequent experiments, we determined the effect of cholesterol depletion on cAMP responses indexed with a different biosensor engineered around the cAMP binding domain from type 2 exchange protein activated by cAMP (Epac2). As this sensor lacks the normal tethering sequence found in the full-length Epac2 protein, it accesses the cytosolic compartment of the cardiac myocyte and reports cAMP signals that are qualitatively distinct from those indexed by the PKA-based sensor [30,41]. In control ARVM expressing the Epac2 probe, exposure to 10 μM ZNT yielded a 4.3 ± 0.8% increase in FRET (Figs. 3D and F). Although the β2-AR cAMP signal is considered to be localised to its site of production, we are not alone in reporting β2-AR FRET responses to cytosolic cAMP sensors in the adult ventricular cell [3]. An initial concern was that this might reflect ZNT-stimulation of the β1-AR, however this is unlikely to be the case because of the lack of effect of ZNT on *I*_Ca,L_; we have recently shown that β1-AR stimulation with isoproterenol (1 nM), which gives an Epac response equivalent to that seen here with ZNT, increases *I*_Ca,L_ by 15% [23]. Importantly, the Epac FRET response in ARVM treated with MBCD was identical (4.2 ± 0.5%) to that seen in control cells (Figs. 3E and F). Therefore, differences in cAMP signals between control and MBCD-treated ARVM reported by cAMP biosensors exist only in regions of the myocyte where PKA-RII is found. Interestingly, these data mirror our recent observations that β1-ARs show enhanced PKA-probe, but not Epac-probe, responses following MBCD treatment [23]. A significant proportion of β1 ARs is in caveolae [21–23]. The specific effects of MBCD on PKA probe responses to β1- and β2-AR stimulation suggest that caveolar signalling complexes restrain cAMP production selectively in PKA RII domains.

**Fig. 4:**
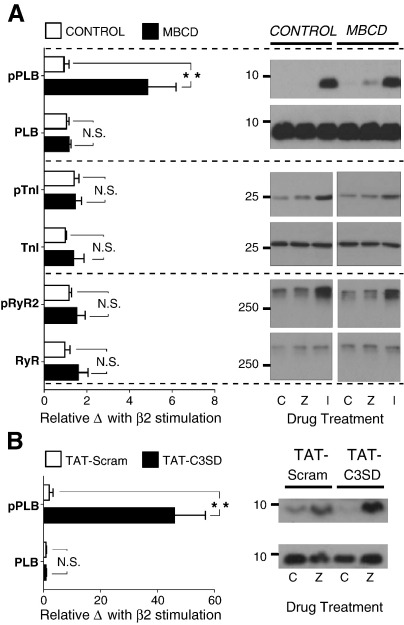
The nascent β2-AR-cAMP signal in cholesterol-depleted ARVM leads to preferential PKA phosphorylation of PLB. A, representative immunoblots from each set are shown on the right. Band intensity measured in the presence of 300 nM CGP and 10 μM ZNT (denoted ‘Z’) was normalised to 300 nM CGP alone (‘C’). Samples from ARVM treated with 100 nM Iso (‘I’) are shown as positive controls for PKA-mediated phosphorylation. Average β2-AR-stimulated change in total and PKA-phosphorylated signals are presented in the bar graph (pPLB, PLB phospho-Ser^16^; pTnI, TnI phospho-Ser^23/24^; pRyR2, RyR2 phospho-Ser^2809^) (n = 6; **P < 0.01 vs. control, N.S. = not significant). B, the right hand panel shows a representative immunoblot showing pPLB and total PLB under basal (300 nM CGP; C) and β2-AR stimulated (10 μM ZNT/300 nM CGP; Z) conditions in TAT-Scram and TAT-C3SD treated ARVM. The bargraph shows pPLB and PLB band intensity measured in ‘Z’ normalised to ‘C’ (n = 3; **P < 0.01 vs. TAT-Scram). All comparisons with Student's t-test.

Given that PKA-RII is found in many different regions of the adult cardiac myocyte, our next aim was to identify which cellular compartments are accessed by the observed ZNT-mediated mobilisation of cAMP in MBCD-treated cells. We addressed this question by measuring PKA-dependent phosphorylation of 3 distinct targets following β2-AR stimulation. The SR protein PLB is found in non-junctional SR and is phosphorylated by PKA at Ser^16^, a modification which relieves its inherent inhibition of SR Ca^2+^-ATPase, SERCA, and thus promotes re-uptake of Ca^2+^[42]. The major SR Ca^2+^ release channel in ARVM, RyR2, is found predominantly within junctional SR and can be positively modulated by PKA phosphorylation at Ser^2809^ and Ser^2030^[43,44]. At the myofilaments, PKA exerts a Ca^2+^ de-sensitising effect *via* dual phosphorylation of TnI at Ser^23^ and Ser^24^[45]. These three proteins can act as spatial references to map the pattern of PKA phosphorylation. Representative immunoblots in Fig. 4A show that application of 100 nM isoproterenol markedly increases the phospho-antibody signal intensity for the 3 targets in both control and MBCD-treated ARVM. However, for all targets, no change in phosphorylation status was detected between basal and β2-AR-stimulated conditions in control ARVM (P > 0.05). This was not surprising given the lack of effect of ZNT on the magnitude of [Ca^2+^]_i_ transients and shortening in these cells. By contrast, β2-AR activation in cholesterol-depleted ARVM produced a marked increase in Ser^16^ phosphorylated PLB (pPLB), in agreement with the effects of MBCD on [Ca^2+^]_i_ transient amplitude and t_0.5_ decay described in Fig. 1. We detected no increase in phosphorylation of TnI or RyR2 (at Ser^2809^) in MBCD-treated cells. In the latter case this could be related to previous reports of a high background phosphorylation at Ser^2809^ in the absence of any cAMP-raising stimulus [46]. We therefore repeated these experiments with an antibody against RyR phosphorylated at Ser^2030^, but we were unable to detect a phospho-RyR signal in either group of cells, even following treatment with 100 nM isoproterenol (data not shown). From the results displayed in Fig. 4A, we surmise that the emergence of β2-AR responsiveness after cholesterol depletion is linked to a discrete pattern of PKA activity in a cellular compartment that contains PLB.

Up to this point, cholesterol depletion was employed to disrupt lipid rafts which include, but are not limited to, caveolae. In order to determine whether observed effects of MBCD could be ascribed specifically to effects on caveolae, we designed a cell-permeable peptide (TAT-C3SD) which competes with endogenous C3SD for the same intracellular binding partners [47,48]. A second peptide (TAT-Scram), containing a scrambled sequence of C3SD was used as a negative control. We addressed the question of whether TAT-C3SD has any effect on β2-AR-mediated cAMP signalling in ARVM using pPLB as an index of discrete PKA activity revealed by MBCD treatment. Myocytes treated with TAT-Scram peptide display a low level of Ser^16^ pPLB after β2-AR activation which is not significantly different from that seen under basal conditions (P > 0.05). By contrast, myocytes treated with TAT-C3SD peptide show a markedly enhanced β2-AR pPLB signal relative to CGP background level (P < 0.01 vs. TAT-Scram; Fig. 4B). We cannot compare the relative magnitude of MBCD and C3SD peptide effects on ZNT-induced pPLB, as analysis of immunoblots in this way is, at best, semi-quantitative. However, the qualitative difference between TAT-Scram and TAT-C3SD treated ARVM in terms of β2-stimulated pPLB is pointedly reminiscent of the difference observed between control and MBCD-treated ARVM shown in Fig. 4A. This supports our claim that MBCD treatment exerts its effects via selectively targeting caveolae.

**Fig. 5:**
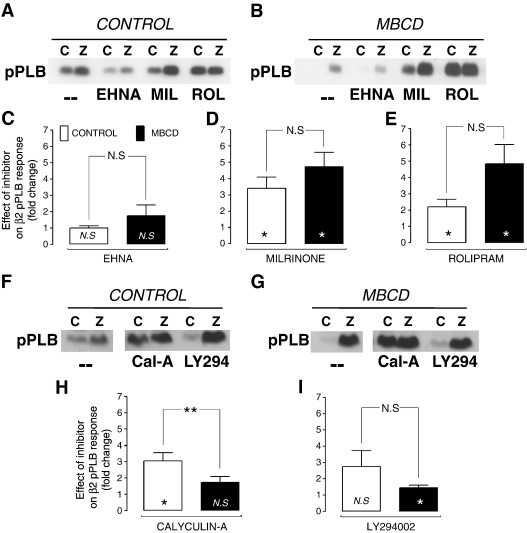
The effect of PDE isoform, PP, and PI3K inhibition on β2-AR-stimulated PKA phosphorylation of PLB. PLB phosphorylated at Ser^16^ (pPLB) was measured in control and MBCD cells, under basal (300 nM CGP; C) and β2-AR stimulated (10 μM ZNT/300 nM CGP; Z) conditions after pre-incubation with the PDE2 inhibitor EHNA (10 μM), PDE3 inhibitor milrinone (MIL, 10 μM), PDE 4 inhibitor rolipram (ROL, 1 μM), PP inhibitor calyculin-A (Cal-A, 50 nM), or the PI3K inhibitor LY294002 (LY294, 10 μM). -- indicates no treatment with inhibitor. Levels of pPLB levels were first normalised to total PLB in the same sample, then these normalised values for the ZNT response (Z) in the presence of inhibitor were divided by normalised pPLB for the ZNT response in the absence of inhibitor. Symbols within bars indicate 1 sample t-test vs 1, whereas symbols associated with connecting line indicate 2 sample t-test between control and MBCD groups (n = 6; N.S. not significant, *P < 0.05; **P < 0.01).

Our next aim was to reconcile data showing a role for PDEs [14] and protein phosphatases (PP) [12] in the compartmentation of β2-AR signals with the mechanism by which caveolae exert their control on this pathway. We were also interested in phosphoinositide 3-kinase (PI3K), which lies upstream of both PDE [49] and PP [12] activation, through scaffolding and kinase-dependent activity respectively. The PDE family is of prime importance in the creation of intracellular gradients of cAMP (see Ref. [50]). Nevertheless, the dependence of PDEs on intact caveolae has never, to our knowledge, been explored in cardiac cells, although a recent report in hepatocytes suggests that Cav-1 stabilises PDE3B in caveolae [51]. Adult rat cardiomyocytes express predominantly PDE 3 and 4 isoforms (PDE 3A, 4A,B,D), with low levels of PDE2 [14]. We therefore compared the effect of selective inhibition of PDE 2, 3 and 4 on β2-AR signalling in control and MBCD-treated ARVM. Our premise was that if caveolar control was dependent on PDE activity, then MBCD treatment would diminish the impact of PDE inhibition on the β2-AR response. The level of pPLB was measured in ARVM pre-treated for 15 min with EHNA, milrinone or rolipram (to inhibit PDE 2, 3 or 4 respectively) plus CGP, and following subsequent stimulation with ZNT (using the same regime as outlined for experiments in Fig. 4A). The effects of the inhibitors on basal phosphorylation (i.e. in the presence of CGP alone) in the 2 groups of cells are shown in Supplementary Fig. 2. There was no significant difference in the effect of any inhibitor on basal pPLB between control and MBCD cells. Figs. 5 (C–E) shows the effect of each PDE inhibitor on the β2-AR pPLB response, i.e. the fold-change in pPLB with inhibitor plus ZNT over ZNT alone. In control ARVM (i.e. cells with intact caveolae), inhibition of PDE3 or PDE4, but not PDE2, significantly enhanced phosphorylation of PLB during ZNT application (P < 0.05). There was no significant difference in the effect of any inhibitor of PDE on β2-AR stimulated pPLB between control and MBCD-treated cells. These data suggest that PDE3 and 4 make a significant contribution to compartmentation of the β2-AR cAMP signal in control cells, which is consistent with previous work showing that PDE3/4 inhibition increases the size and duration of the β2-AR cAMP and activated PKA signals (e.g.[4,14]). However, as MBCD treatment did not *diminish* the effect of PDE inhibition on β2-AR pPLB responses, cAMP compartmentation by PDE 3 or 4 at the level of the SR cannot be reliant on intact caveolae/rafts.

Next, using the same approach, we tested the effect of inhibitors of PP (calyculin-A, which inhibits PP1 and PP2A) and PI3K (LY294002) on the ZNT-induced phosphorylation status of PLB. In control myocytes there was a trend for both calyculin-A and LY294002 to enhance phosphorylation of PLB during β2-AR stimulation, but this only attained significance for calyculin-A (P < 0.05) (Figs. 5H, I). In MBCD-treated myocytes, the effect of phosphatase inhibition was significantly reduced (P < 0.01) compared with control cells. We are aware that effects of inhibitors on basal phosphorylation of PLB may affect the response to β2-AR stimulation. However, this cannot account for the reduced effect of calyculin-A in MBCD cells as basal phosphorylation was not different in MBCD cells treated with this drug compared with controls (Supplementary Fig. 2). The fact that calyculin-A enhances β2-AR pPLB responses in control cells and that its effects are diminished by MBCD implies a common pathway for caveolar- and phosphatase-compartmentation of the β2-AR signal. Together these data suggest that phosphatases make a major contribution to compartmentation of the β2-AR cAMP signalling, and that this mechanism is dependent on intact caveolae.

**Fig. 6:**
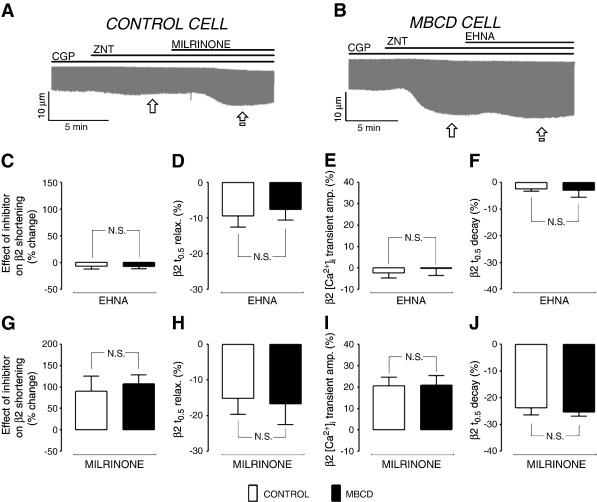
Cholesterol depletion does not alter the effect of PDE2 or PDE3 inhibition on β2-AR-regulated ARVM contractile properties. The experimental design employed to measure responses to PDE isoform inhibition is illustrated by representative traces from control (A) and MBCD-treated (B) cells during perfusion with various pharmacological agents (CGP, 300 nM; ZNT, 10 μM; EHNA, 10 μM; milrinone, 10 μM). The effect of inhibitors was assessed as the % change in each parameter at steady state (i.e. between  and ). Mean data for the effect of PDE2 (10 μM EHNA) and PDE3 (10 μM milrinone) inhibition on the β2-AR induced response (shortening, [Ca^2+^]_i_ transient amplitude, t_0.5_ relaxation, and t_0.5_ [Ca^2+^]_i_ transient decay) are shown in C–F and G–J, respectively (n = 8–11 cells, N.S. = not significant; Student's t-test).

To complement data shown in Fig. 5, we looked next at the effects of inhibiting PDEs, PP and PI3K on control and MBCD-treated myocyte function, which effectively indexes the sum of all phosphorylation events in the cell. Inhibitors were applied after the response to ZNT had stabilised (or after 5 min in the case of control cells yielding no response), as illustrated in representative traces (Figs. 6A, B). The impact of the isoform-specific PDE inhibitors on the steady-state β2 AR response is shown in Figs. 6 (C–J). In control cells, inhibition of PDE2 with EHNA had no significant effect (P > 0.05 vs. 0) on shortening or [Ca^2+^]_i_ transient amplitude responses to β2-AR stimulation (Figs. 6C, E), but slightly enhanced (P < 0.05 vs. 0) effects of β2-AR stimulation on relaxation and decay kinetics (Figs. 6D, F). The effect of EHNA was identical in MBCD-treated AVRMs. The PDE3 inhibitor milrinone significantly enhanced (P < 0.05 vs. 0) ZNT-induced changes in shortening, [Ca^2+^]_i_ transient amplitude, *t*_0.5_ relaxation and *t*_0.5_ decay in control cells; quantitatively similar effects were seen in MBCD-treated ARVM (P > 0.05 between groups). The identical effects of PDE2 or 3 inhibition in control vs MBCD cells suggest that PDE2/3 inhibition and MBCD exert independent influences on β2-AR cell function. The same comparison between control and MBCD-treated cells was not possible with PDE4 inhibition as application of rolipram (200 nM – 1 μM) elicited spontaneous contractile events in MBCD cells (see Supplemental Fig. 3), indicating a level of Ca^2±^ dysregulation that precluded analysis of electrically evoked [Ca^2±^]_i_ transients. These synergistic effects of cholesterol depletion and PDE4 inhibition warrant further investigation. However, for the purpose of the present investigation, these data suggest that the functional effects of rolipram are not *attenuated* in MBCD cells vs controls, as we report for pPLB.

**Fig. 7:**
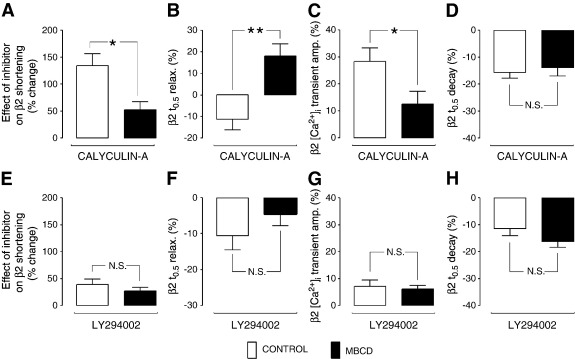
Cholesterol depletion attenuates the effect of PP, but not PI3K, inhibition on β2-AR-regulated ARVM contractile properties. Mean data showing the effect of PP inhibition (50 nM calyculin-A) and PI3K inhibition (10 μM LY294002) on the β2-AR induced response (shortening, [Ca^2+^]_i_ transient amplitude, t_0.5_ relaxation, and t_0.5_ [Ca^2+^]_i_ transient decay) are shown in A–D and E–H, respectively. (N.S. = not significant; *P < 0.05; **P < 0.01; Student's t-test).

Figs. 7 (A–D) show the impact of inhibition of PP on functional responses to β2-AR stimulation in control and MBCD-treated ARVMs. Exposure of control ARVM to calyculin-A markedly enhanced ZNT-stimulated cell shortening and [Ca^2+^]_i_ transient amplitude (by 134 ± 22 and 28.2 ± 5.1% respectively), and further decreased *t*_0.5_ relaxation and *t*_0.5_ transient decay (by 11.3 ± 4.9 and 15.7 ± 2.2% respectively). The effects of calyculin-A on shortening and [Ca^2+^]_i_ transient amplitude were significantly attenuated (P < 0.05) in MBCD cells compared with controls (to 51.9 ± 15.4% and 10.8 ± 4.4% respectively)(Figs. 7A, C). Furthermore, calyculin-A reversed the positive lusitropic effect of β2-AR stimulation in MBCD-treated ARVM (18.0 ± 5.7% increase in *t*_0.5_ relaxation) (Fig. 7B), but this was not associated with a corresponding change in [Ca^2+^]_i_ transient decay (Fig. 7D). The attenuation of calyculin-A effects on β2-AR responses in MBCD cells vs. controls confirms a caveolae-dependent role for PP in limiting β2-AR signals. Although we failed to detect a difference in the effect of calyculin A on *t*_0.5_ transient decay between control and MBCD AVRM, we are reluctant to interpret this as evidence that caveolar phosphatases are not active in the PLB compartment, given that calyculin-A has significantly less effect on phospho-PLB in MBCD cells compared with controls (Fig. 5H). This apparent discrepancy may arise because *t*_0.5_ transient decay indexes more than SR Ca^2+^ uptake control by PLB; it is a composite of many mechanisms that alter [Ca^2+^]_i_, including SR Ca^2+^ uptake, SR Ca^2+^ leak and sarcolemmal Ca^2+^ extrusion.

The effect of PI3K inhibition on functional responses to β2-AR stimulation is shown in Figs. 7 (E–H). In control cells, the application of LY294002 significantly enhanced (P < 0.05 vs. 0) cell shortening, [Ca^2+^]_i_ transient magnitude, the rate of relaxation and the rate of transient decay in response to ZNT. None of the LY294002-induced changes in cell shortening and [Ca^2+^]_i_ transient parameters was significantly different between control and MBCD-treated groups (P > 0.05 for all comparisons), suggesting that PI3K control of the β2 AR signal is not dependent on caveolae.

Together these functional data suggest that PDE3, PI3K and phosphatases make a significant contribution to compartmentation of β2 cAMP signalling in the control AVRM, which is in agreement with previous reports [12,14]. We were unable to confirm a contribution from PDE4 seen in experiments using pPLB as our index of the cAMP signal because of dysregulation of [Ca^2+^]_i_ when PDE4 was inhibited in MBCD-treated cells. Comparison of datasets using pPLB and cell function as indices of cAMP signals demonstrates that we were only able to detect a significant contribution from PI3K to restriction of the β2-AR cAMP signal using functional endpoints. The disparity between these results may reflect the fact that PI3K effects are not mediated through changes in a cAMP compartment that contains PLB. However, an alternative explanation is that the statistical power of our functional experiments is higher because we were able to measure the effect of ZNT alone and ZNT plus inhibitor *in the same cell.*

According to our premise that if a particular regulatory element were dependent on intact caveolae, the effect of inhibition of this element would be reduced in MBCD-treated cells, these data identify, for the first time, phosphatases as caveolae-dependent factors which compartmentalise β2-AR signalling.

**Fig. 8:**
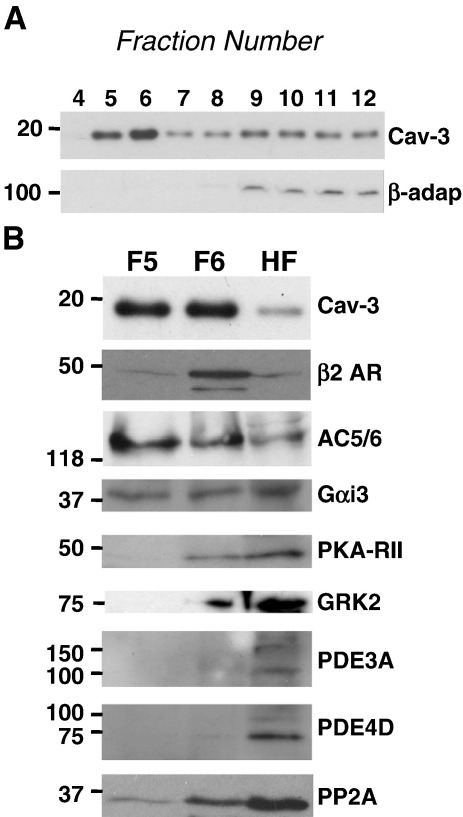
Distribution of key proteins associated with cAMP compartmentation in sucrose gradient fractions of ARVM membranes. A, representative immunoblots for Cav-3 and β-adaptin (β-adap) in sucrose gradient fractions 4 to 12 of ARVM. Buoyant fractions 5 and 6 (F5 and F6) are designated caveolar fractions because of enrichment in Cav-3, and exclusion of β-adaptin. B, F5, F6, and a 1:1:1:1 mix of fractions 9–12 (heavy fractions, HF) were immunoblotted for the presence of Cav-3, β2-AR, AC5/6, Gαi3, PKA-RII, GRK2, PDE3A, PDE4D, and PP2A.

Although caveolar-dependent phosphatases contribute to compartmentalisation of β2-AR signalling in the ventricular cell, this cannot be the only mechanism by which caveolae act in this regard because, as shown using FRET-based biosensors, disruption of caveolae with MBCD also increases cAMP levels in a PKA RII compartment. The level of cAMP in the cell will represent a balance between cAMP production (by AC) and degradation (by PDEs). Our data suggest that caveolae do not contribute to compartmentalisation of cAMP through effects on PDE activity, therefore we conclude that caveolae must regulate cAMP production by AC. β2-ARs couple sequentially to Gs and Gi proteins [52]. β2-AR cAMP generation can be modulated through changes in Gαs and Gαi coupling (which respectively activate and inhibit AC 5/6) [53] and through receptor internalisation (which curtails cAMP production) [54]. A common mechanism which controls both β2-AR-G protein coupling and internalisation is receptor phosphorylation. Receptors can be phosphorylated by PKA [55] and G protein receptor kinase (GRK [56]). PKA and GRK phosphorylation of the β2-AR shifts coupling from Gs to Gi [55,57] and GRK-dependent phosphorylation recruits β-arrestin to the receptor [58,59] which prevents receptor-G protein coupling and triggers receptor internalisation. One attractive model for caveolar restriction of β2-AR cAMP production is that the caveolar compartment facilitates PKA- and/or GRK-dependent phosphorylation of the receptor. Therefore, in order to gain insight into *how* caveolae control cAMP production and phosphatase activity, we performed sucrose gradient fractionation to look at the distribution of relevant signal components between caveolar and non-caveolar domains in control cells. As shown in Fig. 8, the buoyant Cav-3 containing fractions (5 and 6) contain the majority of the β2-AR and AC 5/6, a significant proportion of Gαi, PKA RII and PP2A, and a minor component of GRK2. The main PDE3 and 4 isoforms expressed in the rat heart, PDE3A and PDE4D, were found almost exclusively in non-caveolar fractions. This membrane distribution of key components of the β2-AR pathway provides a potential framework for caveolar control of cAMP production by facilitating PKA/GRK2 phosphorylation of the β2-AR and consequent receptor-Gi coupling. This, in turn, could have consequences for regulation of phosphatases in the SR compartment (see Discussion).

## 4 Discussion

Compartmentation of cAMP is a fundamental strategy which allows cells to orchestrate complex receptor signalling with a single second messenger. Over the last decade a body of evidence has accumulated which suggests that Gi coupling, protein phosphatases and PDEs play essential roles in creating the distinct β2-AR cAMP-dependent signature in the ventricular myocyte. At the same time, a number of groups, including our own, have shown that compartmentation of the β2-AR pathway is dependent on the caveolar microdomain.

Here, for the first time, we reconcile these data by showing how the spatial characteristics of the cAMP signal and PKA-dependent phosphorylation following β2-AR stimulation are dependent on caveolae, and how PDEs, PP and PI3K contribute to caveolar control.

The 2 essential aspects of our experimental design were to use the most physiologically relevant model (adult ventricular myocytes expressing endogenous β-AR) and to achieve selective activation of the β2-AR (through use of the β2-AR agonist ZNT with a selective β1 antagonist CGP). As we predicted our approach has revealed discrepancies with other studies using neonatal myocytes and and/or other drugs to activate the β2-AR.

In the present study, control ARVM showed no change in shortening, [Ca^2+^]_i_ transient amplitude or *I*_Ca,L_ in response to β2-AR stimulation with 10 μM ZNT in the presence of 300 nM CGP. This is consistent with other work in these cells using this combination of ZNT and CGP [1,60]. We show here that disruption of caveolae through cholesterol depletion with MBCD allows β2-AR stimulation with ZNT to elicit robust inotropic and lusitropic responses which can be ascribed to effects on SR Ca^2+^ uptake and/or release, with no contribution from *I*_Ca,L_ .

These data contrast with our previous work showing a small *I*_Ca,L_ response to β2-AR stimulation with the β2-AR agonist salbutamol (with atenolol) in control cells that is potentiated by MBCD treatment [27]. The difference between the effects of zinterol and salbutamol could arise because of ligand-selective agonism, whereby downstream Gs/Gi coupling is determined by a particular ligand's stereochemical properties [61,62]. However, in the context of our recent report that MBCD treatment can potentiate the *I*_Ca,L_ response to sub-maximal β1-AR stimulation [23], a more likely explanation is that salbutamol (with atenolol) produces a small, but significant, activation of the β1-AR pathway.

A key question for the present study was how the functional effects of β2-AR stimulation with zinterol in MBCD-treated cells relate to changes in the spatial characteristics of the cAMP signal and the resulting PKA-dependent phosphorylation. Both PDE and PP have been linked with control of the β2-AR signal, and compartmentation may occur at the level of cAMP and protein phosphorylation independently. We used FRET-based biosensors that allow measurement of cAMP in real-time in 2 different cellular compartments to show that disruption of caveolae with MBCD reveals a nascent β2-AR stimulated rise in cAMP specifically in PKA-RII regions of the cell. This rise in cAMP in a PKA-RII compartment is associated with a selective increase in phosphorylation of the SR protein PLB, which suggests that the increase in cAMP occurs specifically in the PLB-containing compartment of the SR. Indeed, this accords with the fact that MBCD enhances the rate of [Ca^2+^]_i_ transient decay in response to β2-AR stimulation. However, at this stage we cannot exclude the possibility that changes in PP activity could also contribute to altered phosphorylation of PLB.

Again, these experiments highlight subtle differences in the subcellular effects of different β2-AR agonist/β1-AR antagonist pairs. In the present study, of our 3 PKA targets selected as spatial reference points in the cell (PLB, RyR and TnI), only PLB was phosphorylated by β2-AR in MBCD-treated cells. However, with salbutamol (plus atenolol), β2 stimulation also enhances TnI phosphorylation in MBCD treated cells [9]. Again, a simple explanation is that this represents effects mediated through stimulation of a β1-AR component.

We have shown that the cholesterol-depleting agent MBCD markedly reduces caveolar density [31], but this does not guarantee that MBCD effects on the β2-AR pathway are mediated through caveolae, as cholesterol is also enriched in non-caveolar lipid rafts, and additionally contributes to physical properties (thickness, fluidity) of non-raft membranes [63]. However, here we show that a cell permeable peptide, which severs the interaction of Cav-3 with its normal binding partners, mimics the effect of MBCD on phosphorylation of PLB following β2 stimulation. Thus we are confident that the MBCD effects we report are mediated through the caveolar microdomain.

Having established phosphorylation of PLB as a robust index of the β2-AR signal seen when caveolae are manipulated by MBCD or TAT-C3SD, we were able to test how the various components linked with compartmentalisation of this pathway are involved in caveolar control. These data show that, whilst PDE3, PDE4 and PP contribute to compartmentation of the β2-AR signal in control cells, only the PP effect is dependent on caveolae. When we take changes in [Ca^2+^]_i_ and contractility together as an index of the sum of *all* phosphorylation events occurring following β2-AR stimulation, we see an additional contribution from PI3K in control cells, but the pattern of response to all inhibitors between control and MBCD-treated cells is maintained. Together these data show quite clearly that the control of the β2-AR signal by PDEs is independent of caveolae, whilst control by PP is dependent on an intact caveolar domain.

Data from effects of calyculin-A (Figs. 5 and 7), and measurement of cAMP biosensor responses (Fig. 3), strongly support the view that caveolae exert their compartmentalisation of β2-AR signals through effects on both cAMP production and phosphatase activity. With regard to the former effect, we were particularly interested in the possibility that this could be regulated via phosphorylation of the β2-AR by either PKA or GRK. Receptor phosphorylation has been shown to affect the kinetic characteristics of the β2-AR signal [64] and it has been suggested recently that it is the transient nature of the β2-AR-dependent cAMP signal that determines its spatial properties (e.g. lack of propagation to the SR) [4]. Our PKA-probe data hint at this change from transient to sustained cAMP signals when caveolae are disrupted with MBCD (Figs. 3A, B). We were unable to measure β2-AR phosphorylation directly, as antibodies for the PKA- and GRK- phosphorylated receptors are raised against human β2-AR and do not recognise the rat β2-AR expressed at endogenous levels (data not shown). Instead we looked at the distribution of relevant components between caveolar and non-caveolar membrane fractions. As others have reported previously in the adult myocyte, we see the majority of β2-AR and AC 5/6 in caveolae-containing fractions, along with a significant proportion of Gαi and PKA RII [20,22]. GRK2, the predominant cardiac isoform [65], was found mostly in non-caveolar fractions, with only a small component in caveolar rafts, as has been reported previously in neonatal cells [35]. This observed pattern of membrane distribution of components could facilitate PKA- (and perhaps GRK2-) dependent phosphorylation of the β2-AR and thereby coupling between β2 and Gi. Indeed we have previously shown that abolition of Gi signalling with pertussis toxin mimics the effect of MBCD on the inotropic response to β2-AR stimulation [27], suggesting that caveolae control is exerted through Gi.

The demonstrated role for phosphatases in the compartmentation of the β2-AR response could be linked with caveolae as a β2-Gi compartment described above. In the ARVM, pertussis toxin enhances phosphorylation of PLB induced by zinterol [12] and has equivalent non-additive effects to calyculin-A on β2-AR mediated inotropy [11]. This suggests that PP activation is Gi-dependent. PP2a is found predominantly at the sarcolemma [66], where a proportion is present in caveolae (as we show here) in a complex with Cav-3 [22]. However, our data suggest that the role of caveolae in phosphatase-dependent restriction of β2-AR signalling is exerted at the level of SR (PLB). Interestingly, a significant proportion of PP1, which is also inhibited by calyculin-A, is found in SR membranes [66]. PP1 is under the control of PP inhibitor 1 (I-1) [67] and PKA-dependent phosphorylation of I-1 enhances its inhibitory effect on PP1, keeping PP activity low [68]. We speculate that disrupting caveolae enhance cAMP production (by effects on Gi-β2-AR coupling); this in turn engenders a more sustained (and propagating) activation of PKA which increases phosphorylation of PLB both directly, and by dampening PP1 activity via I-1. It is interesting to note that PP1 isoforms are differentially distributed in longitudinal SR and junctional SR [66] which could account for the differential effects of disrupting caveolae on phosphorylation of PLB and RyR that our data suggest. This model of compartmentation by caveolae is represented schematically in Supplementary Fig. 4.

## 5 Conclusions

The present study has shown for the first time that caveolae compartmentalise the β2-AR pathway through effects on cAMP production and phosphatase activity, which can be ascribed to facilitation of receptor-Gi coupling. Although the β2-AR pathway may contribute little to the contractile effects of sympathetic stimulation in the healthy heart, it is likely to play a significant role (through modulation of β1 responsiveness [69], and anti-apoptotic signalling [70]) in heart failure. Insight into the caveolar control of β2-AR signalling will impact on our understanding of changes that take place in the failing heart, given that heart failure is associated with increased β2/β1-AR ratio [71], higher Gi expression [72] and diverse changes in caveolae [73–75].

The following are the supplementary materials related to this article.

Supplementary Fig. 1 Density of caveolae does not change during 48 h in culture. Caveolae were visualised using transmission EM in freshly isolated ARVM (CONTROL) and ARVM that had been maintained in culture for 48 h. Caveolae were defined as either 50–100 nm flask-shaped invaginations in the surface membrane or sealed circular vesicles of the same size within 300 nm of the sarcolemma. Caveolar density was similar between groups; 0.96 ± 0.13 per μm membrane for control and 0.86 ± 0.11 per μm membrane for cultured ARVM (n = 9–11 cells from 3 hearts; N.S. = not significant; Student's t-test).

Supplementary Fig. 2 Effects of PDE, PP and PI3K inhibitors on PLB phosphorylation under basal conditions. Phospho-Ser^16^ PLB (pPLB) was expressed relative to total PLB in the sample. A, no inhibitor (−−), EHNA (10 μM), milrinone (MIL, 10 μM), rolipram (ROL, 1 μM). B, no inhibitor (−−), calyculin-A (Cal-A, 50 nM), LY294002 (10 μM). *P < 0.05, ***P < 0.001 vs (−−) within control or MBCD group. There was no significant difference in the effect of any inhibitor between control and MBCD-treated ARVM. Two-way ANOVA with pair-wise comparison by Holm-Sidak (n = 6).

Supplementary Fig. 3 PDE4 inhibition causes dysregulation of [Ca^2+^]_i_ in MBCD-treated cells. Shown are representative myocyte shortening (A) and [Ca^2+^]_i_ transient (B) traces from a MBCD-treated ARVM perfused with 300 nM CGP alone (CGP), 300 nM CGP plus 10 μM ZNT (CGP + ZNT), and finally 300 nM CGP plus 10 μM ZNT plus 200 nM rolipram (CGP + ZNT + ROL). Unstable and dysfunctional contraction is matched with aberrant [Ca^2+^]_i_ transients in the CGP + ZNT + ROL traces.

Supplementary Fig. 4 Schematic representation of the mechanisms which govern compartmentation of β2-AR signalling by caveolae. We indicate that β2-AR-containing caveolae are in relatively close proximity to regions of the longitudinal SR containing PLB. Recent evidence promotes the inclusion of PLB in a macromolecular complex with PP1, PP inhibitor-1 (I-1), SERCA2, and PKA, orchestrated by long isoforms of AKAP18 (δ and γ) [2–4]. Under normal circumstances, activation of the β2-AR leads to a transient stimulation of AC *via* coupling to Gs; the resulting cAMP/PKA signal may reach PLB, but a counterbalancing level of PP1 activity precludes a functional response (A). In addition, a PKA-induced switch in β2-AR coupling from Gs to Gi abrogates cAMP production, cutting the signal off at the source (B). After disruption of caveolae, a stronger and more propagating cAMP signal tips the balance of PLB phosphorylation/dephosphorylation in favour of phosphorylation (C). The more propagating cAMP signal could also give rise to PKA-dependent phosphorylation and activation of I-1 (D). This would result in inhibition of PP1 and a further imbalance of the kinase/phosphatase activity ratio. As a result, PLB loses its tight association with, and ability to inhibit, SERCA2, so Ca^2+^ re-uptake into the SR is stimulated. EXT., extracellular; INT., intracellular; SR; sarcoplasmic reticulum.

Supplementary material Supplement R1 FINAL.

## Disclosures

None declared.
