# Supplementary material for: Caveolae compartmentalise β2-adrenoceptor signals by curtailing cAMP production and maintaining phosphatase activity in the sarcoplasmic reticulum of the adult ventricular myocyte
Source: J Mol Cell Cardiol. 2012 Feb;52(2):388–400. doi: 10.1016/j.yjmcc.2011.06.014 (PMC3270222; doi:10.1016/j.yjmcc.2011.06.014)
Supplement: Supplementary Fig. 1 — Density of caveolae does not change during 48 h in culture. Caveolae were visualised using transmission EM in freshly isolated ARVM (CONTROL) and ARVM that had been maintained in culture for 48 h. Caveolae were defined as either 50–100 nm flask-shaped invaginations in the surface membrane or sealed circular vesicles of the same size within 300 nm of the sarcolemma. Caveolar density was similar between groups; 0.96 ± 0.13 per μm membrane for control and 0.86 ± 0.11 per μm membrane for cultured ARVM (n = 9–11 cells from 3 hearts; N.S. = not significant; Student's t-test). [file mmc1.doc]

**Methods**

*Electron Microscopy*

Caveolae were visualised by electron microscopy as described in Kozera *et al.* [1]. In brief, ARVM were fixed overnight with 2.5% glutaraldehyde, post-fixed using 1% osmium tetroxide and dehydrated through an ethanol series. Myocytes were embedded in araldite, sectioned and stained with 20% uranyl acetate (30 min) followed by Reynolds lead citrate (30 min). Sections were visualised using a JEOL 1200 transmission electron microscope.

*Statistical analysis*

Statistical significance (P<0.05) was determined by Student’s t-test or two-way ANOVA (with pair-wise comparison by Holm-Sidak) as appropriate.

**Figure Legends**

**Supplementary Figure 1**. Density of caveolae does not change over 48 h in culture. Caveolae were visualised using transmission EM in freshly isolated ARVM (CONTROL) and ARVM that had been maintained in culture for 48 h. Caveolae were defined as either 50- 100 nm flask-shaped invaginations in the surface membrane or sealed circular vesicles of the same size within 300 nm of the sarcolemma. Caveolar density was similar between groups; 0.96 ± 0.13 per µm membrane for control and 0.86 ± 0.11 per µm membrane for cultured ARVM (n = 9-11 cells from 3 hearts; N.S. = not significant; Student’s t-test).

**Supplementary Figure 2**. Effects of PDE, PP and PI3K inhibitors on PLB phosphorylation under basal conditions. Phospho-Ser^16^ PLB (pPLB) was expressed relative to total PLB in the sample. **A,** no inhibitor (**--**), EHNA (10 µM), milrinone (MIL, 10 μM), rolipram (ROL, 1 µM). **B,** no inhibitor (**--),** calyculin-A (Cal-A, 50 nM), LY294002 (10 µM). * P<0.05, *** P<0.001 *vs* (--) within control or MBCD group. There was no significant difference in the effect of any inhibitor between control and MBCD-treated ARVM. Two-way ANOVA with pair-wise comparison by Holm-Sidak (n=6).

**Supplementary Figure 3**. PDE4 inhibition causes dysregulation of [Ca^2+^]_i_ in MBCD-treated cells. Shown are representative myocyte shortening (A) and [Ca^2+^]_i_ transient (B) traces from a MBCD- treated ARVM perfused with 300 nM CGP alone (CGP), 300 nM CGP plus 10 μM ZNT (CGP + ZNT), and finally 300 nM CGP plus 10 μM ZNT plus 200 nM rolipram (CGP + ZNT + ROL). Unstable and dysfunctional contraction is matched with aberrant [Ca^2+^]_i_ transients in the CGP + ZNT + ROL traces.

Fluorescence ratio defines fura-2 emission at 510 nm following excitation at 340 and 380 nm. X-axes scale bars indicate 2 s.

**Supplementary Figure 4. Schematic representation of the mechanisms which govern compartmentation of β2-AR signalling by caveolae**. We indicate that β2-AR-containing caveolae are in relatively close proximity to regions of the longitudinal SR containing PLB. Recent evidence promotes the inclusion of PLB in a macromolecular complex with PP1, PP inhibitor-1 (I-1), SERCA2, and PKA, orchestrated by long isoforms of AKAP18 (δ and γ) [2-4]. Under normal circumstances, activation of the β2-AR leads to a transient stimulation of AC *via* coupling to Gs; the resulting cAMP/PKA signal may reach PLB, but a counterbalancing level of PP1 activity precludes a functional response (A). In addition, a PKA-induced switch in β2-AR coupling from Gs to Gi abrogates cAMP production, cutting the signal off at the source (B). After disruption of caveolae, a stronger and more propagating cAMP signal tips the balance of PLB phosphorylation/dephosphorylation in favour of phosphorylation (C). The more propagating cAMP signal could also give rise to PKA-dependent phosphorylation and activation of I-1 (D). This would result in inhibition of PP1 and a further imbalance of the kinase/phosphatase activity ratio. As a result, PLB loses its tight association with, and ability to inhibit, SERCA2, so Ca^2+^ re-uptake into the SR is stimulated. EXT., extracellular; INT., intracellular; SR; sarcoplasmic reticulum.

Reference List

[1] Kozera L, White E, Calaghan S. Caveolae act as membrane reserves which limit mechanosensitive I(Cl,swell) channel activation during swelling in the rat ventricular myocyte. PLoS One 2009;4:e8312.

[2] Lygren B, Carlson CR, Santamaria K, Lissandron V, McSorley T, Litzenberg J et al. AKAP complex regulates Ca2+ re-uptake into heart sarcoplasmic reticulum. EMBO Rep 2007;8:1061-7.

[3] Aoyama H, Ikeda Y, Miyazaki Y, Yoshimura K, Nishino S, Yamamoto T et al. Isoform-specific roles of protein phosphatase 1 catalytic subunits in sarcoplasmic reticulum-mediated Ca2+ cycling. Cardiovasc Res 2010.

[4] Singh A, Redden JM, Kapiloff MS, Dodge-Kafka KL. The large isoforms of A-kinase anchoring protein 18 mediate the phosphorylation of inhibitor-1 by protein kinase A and the inhibition of protein phosphatase 1 activity. Mol Pharmacol 2011;79:533-40.
