# Supplementary material for: Caveolae compartmentalise β2-adrenoceptor signals by curtailing cAMP production and maintaining phosphatase activity in the sarcoplasmic reticulum of the adult ventricular myocyte
Source: J Mol Cell Cardiol. 2012 Feb;52(2):388–400. doi: 10.1016/j.yjmcc.2011.06.014 (PMC3270222; doi:10.1016/j.yjmcc.2011.06.014)
Supplement: Supplementary material — Supplement R1 FINAL. [file mmc2.ppt]

## Slide 1
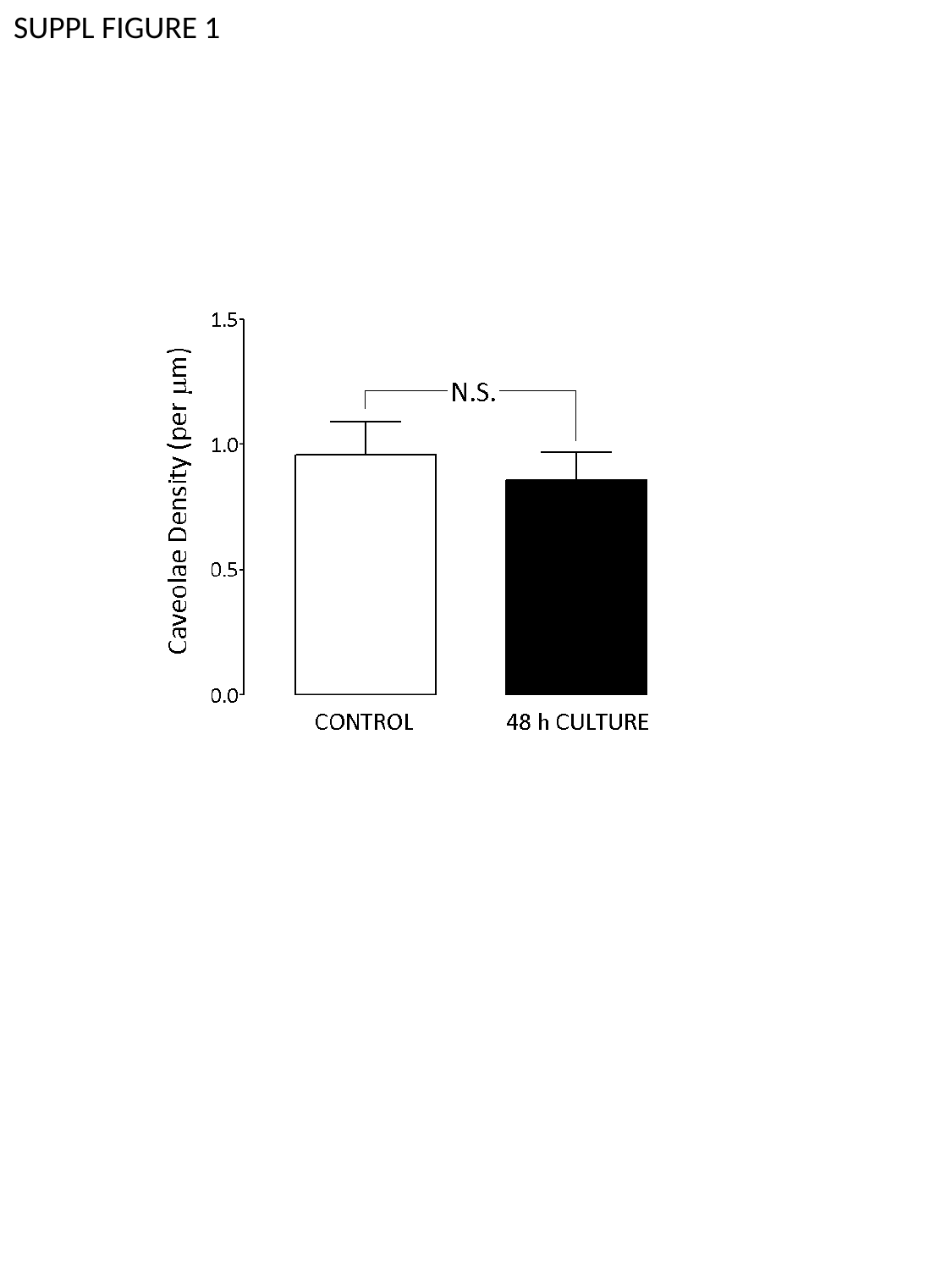

SUPPL FIGURE 1

## Slide 2
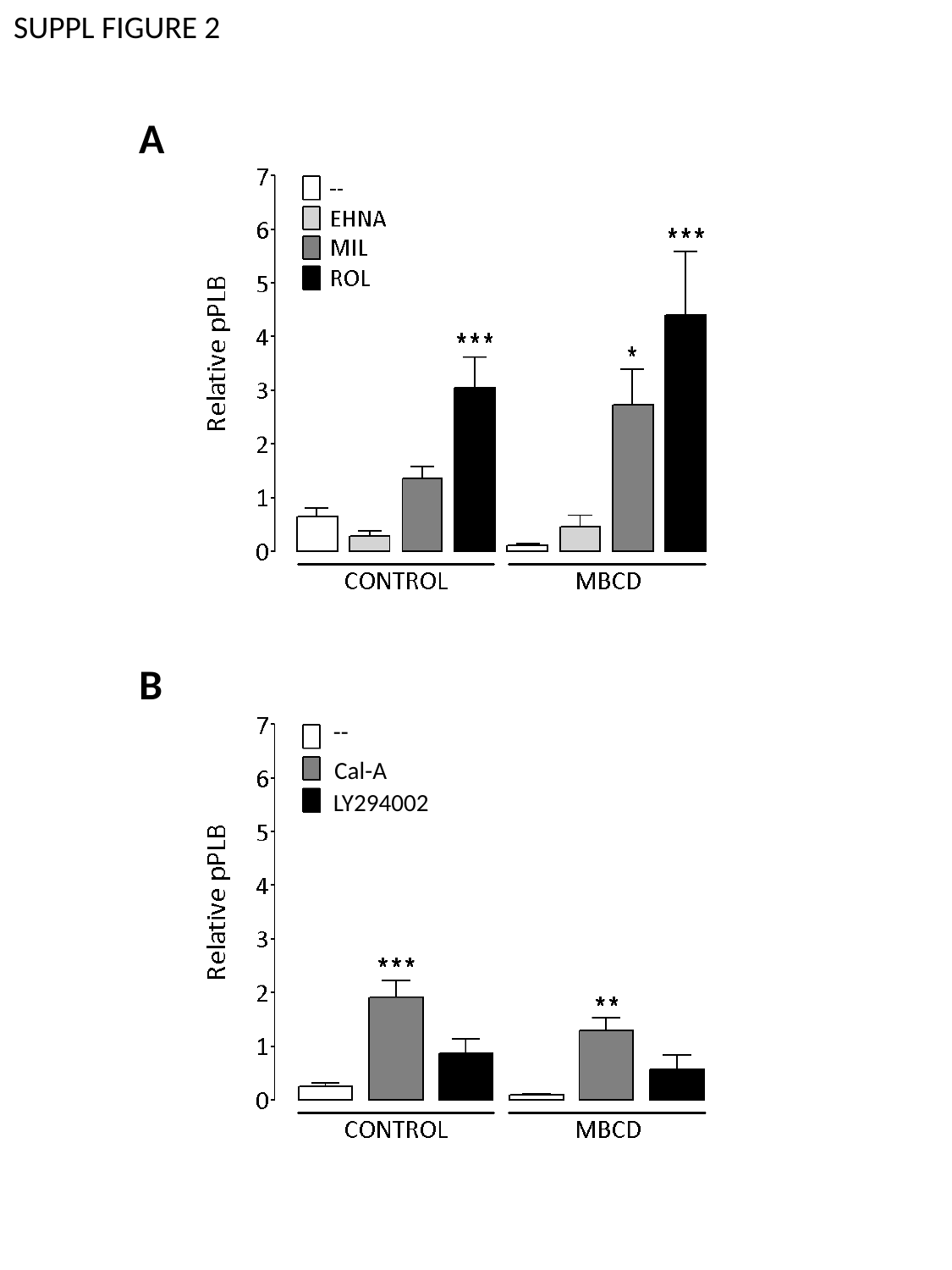

SUPPL FIGURE 2
A
B
--
Cal-A
LY294002

## Slide 3
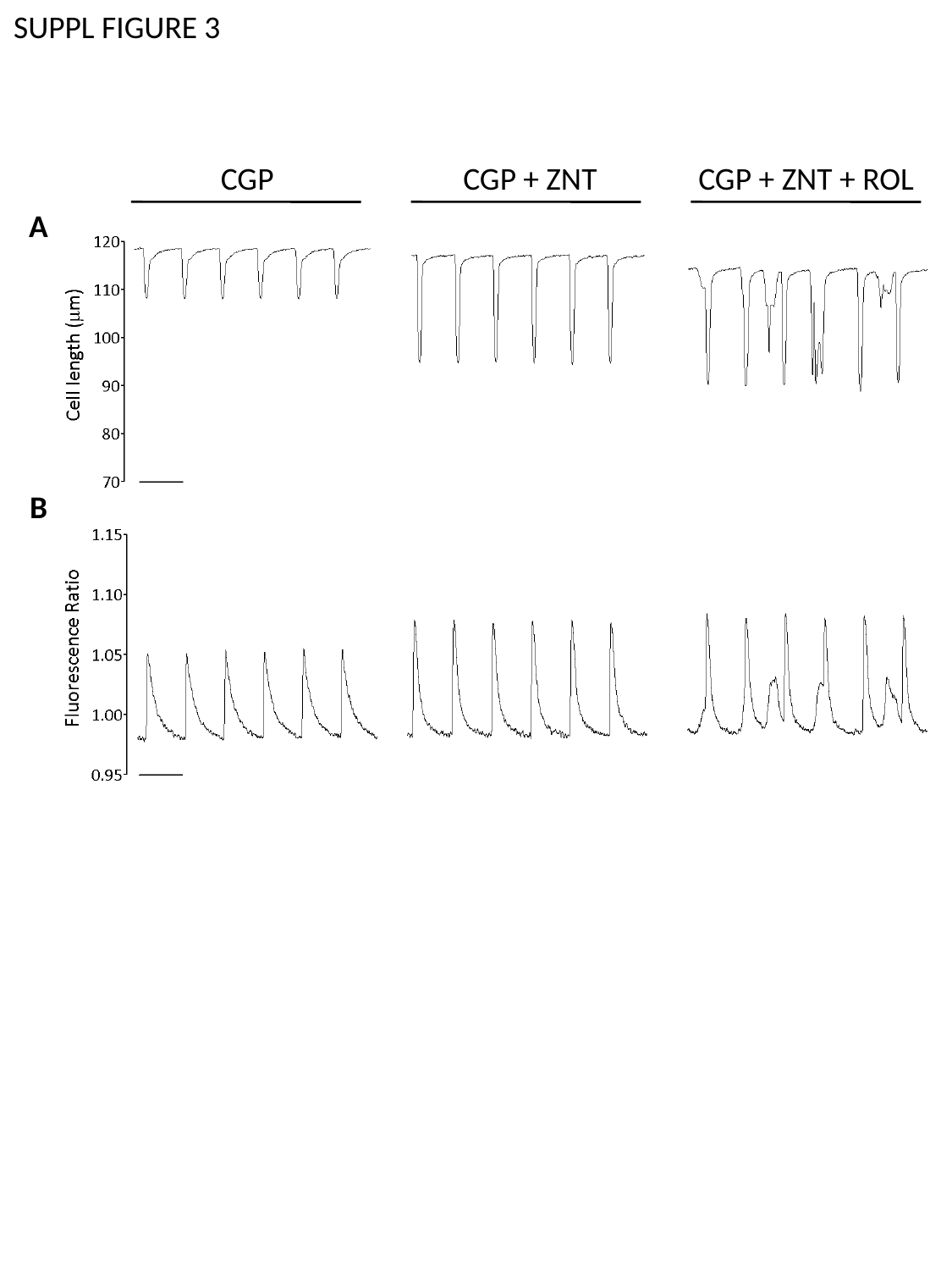

SUPPL FIGURE 3
CGP
CGP + ZNT
CGP + ZNT + ROL
A
B

## Slide 4
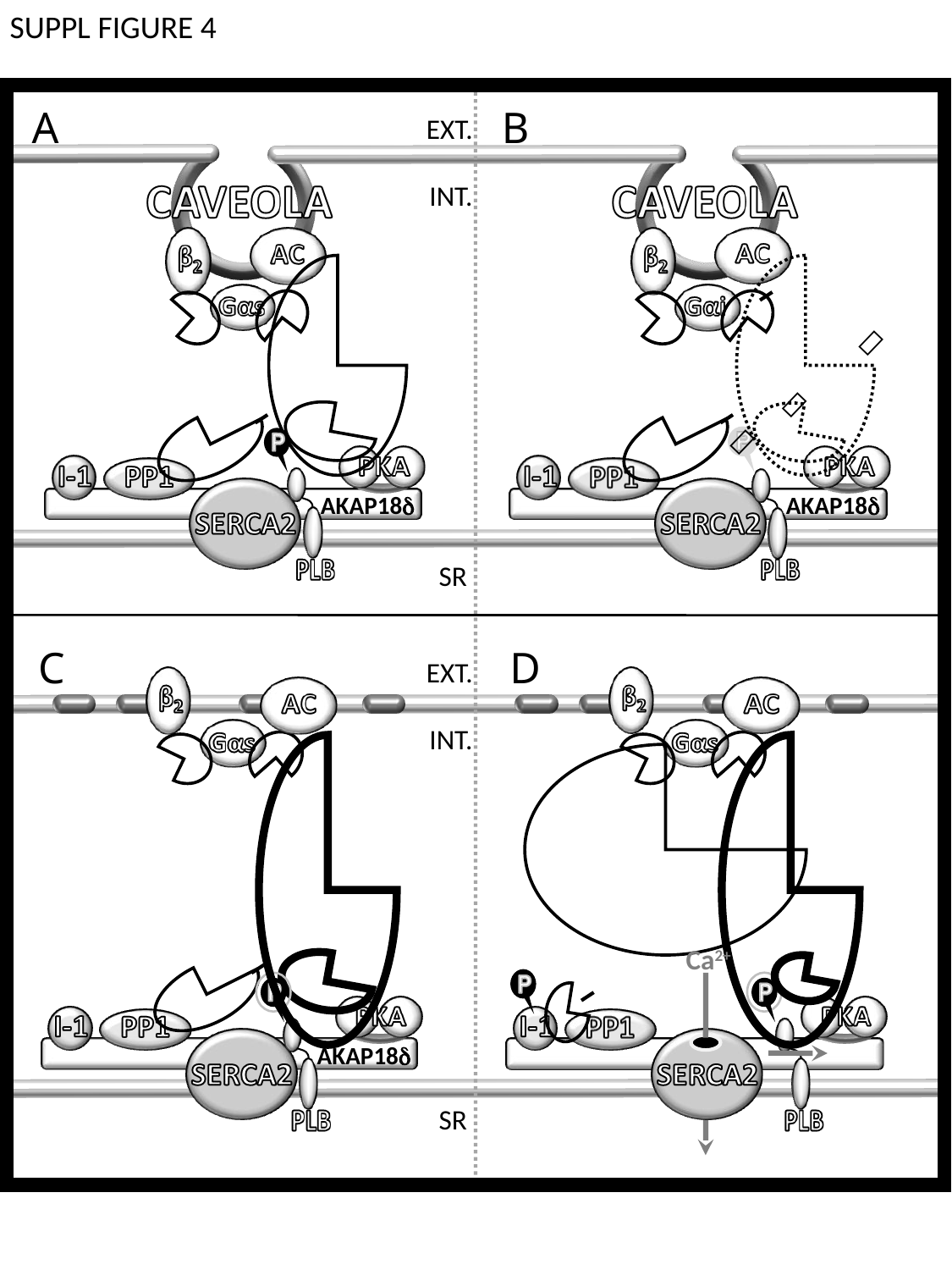

SUPPL FIGURE 4
A
B
EXT.
INT.



AKAP18
AKAP18
SR
C
D
EXT.
INT.
Ca2+
AKAP18
SR
